# Construction and validation of a risk prediction model for vitamin D deficiency in patients with type 2 diabetes mellitus

**DOI:** 10.3389/fnut.2026.1828692

**Published:** 2026-08-05

**Authors:** Ying Zhao, Jieru Chen, Fan Feng, Lixing Yang, Peng Zhang, Xia Cui

**Affiliations:** Department of Pharmacy, Shaanxi Provincial People’s Hospital, Xi’an, Shaanxi, China

**Keywords:** nomogram, risk factors, risk prediction model, type 2 diabetes mellitus, vitamin D deficiency

## Abstract

**Objectives:**

Patients with type 2 diabetes mellitus (T2DM) have a high prevalence of vitamin D deficiency, but convenient and efficient screening tools are lacking in clinical practice. This study aimed to construct and validate a predictive model for vitamin D deficiency risk in T2DM patients based on routine clinical indicators.

**Methods:**

Clinical data were retrospectively collected from 618 T2DM patients hospitalized in the Department of Endocrinology of a tertiary general hospital between January 2024 and December 2024. Patients were randomly divided into a training cohort (*n* = 432) and a validation cohort (*n* = 186) at a ratio of 7:3. LASSO regression was used to screen predictors, and a logistic regression model was constructed to generate a nomogram. The discrimination, calibration, and clinical utility of the model were evaluated using the area under the receiver operating characteristic curve (AUC), calibration curves, and decision curve analysis (DCA), respectively.

**Results:**

The prevalence of vitamin D deficiency in T2DM patients was 71.8%. LASSO regression combined with multivariate logistic regression showed that female (OR = 3.53, 95%CI: 1.99–6.25, *P* < 0.001), elevated triglycerides (TG) (OR = 1.56, 95%CI: 1.15–2.12, *P* = 0.004), elevated glycated hemoglobin (HbA1c) (OR = 1.17, 95%CI: 1.03–1.32, *P* = 0.048), and urinary albumin-to-creatinine ratio (UACR) ≥ 300 mg/g (OR = 9.68, 95%CI: 2.58–29.24, *P* < 0.001) were independent risk factors for vitamin D deficiency, whereas age ≥ 65 years (OR = 0.34, 95%CI: 0.19–0.59, *P* < 0.001) was a potential protective factor. The nomogram model based on these five variables achieved an AUC of 0.7468 (95%CI: 0.6994–0.7942) in the training cohort and 0.7557 (95%CI: 0.6753–0.8362) in the validation cohort. Calibration curves revealed favorable consistency between predicted and actual probabilities (Hosmer–Lemeshow test: training cohort *P* = 0.436, validation cohort *P* = 0.672). DCA indicated net benefit within the clinically defined threshold range.

**Conclusion:**

We developed a nomogram using Gender, Age, TG, HbA1c, and UACR for predicting vitamin D deficiency risk in T2DM inpatients. Internal validation indicated promising performance. While the model may assist in identifying high-risk individuals in hospital settings, its clinical utility and generalizability remain to be confirmed through external validation.

## 1 Background

Diabetes mellitus is a group of metabolic disorders characterized by hyperglycemia caused by absolute or relative insulin deficiency, impaired insulin action, or a combination of both (1). Currently, diabetes is classified according to its underlying pathogenesis, with type 2 diabetes mellitus (T2DM) accounting for 90%–95% of all cases (2). According to the International Diabetes Federation, approximately 415 million adults aged 20–79 years were living with T2DM in 2015, and its prevalence is continuously increasing. The number is projected to rise by an additional 200 million by 2040, and the global disease burden is expected to reach 783 million by 2045 (3, 4). T2DM can affect multiple organ systems, including the pancreas, liver, muscle, kidney, heart, nervous system, intestine, and adipose tissue, thereby inducing a variety of complications and significantly increasing the individual disease burden and pressure on the overall healthcare system.

Vitamin D is an essential fat-soluble vitamin for the human body. By participating in diverse biological processes including calcium and phosphorus metabolism, cell differentiation and proliferation, immune response, and inflammatory regulation, it plays a vital role in maintaining bone health, regulating metabolic homeostasis, preserving cardiovascular function, and preventing tumorigenesis, thus serving as one of the key nutrients for sustaining overall health (5, 6). Whether vitamin D is obtained from dietary intake or synthesized in the skin, it is converted into 25-hydroxyvitamin D [25(OH)D] in the liver. This metabolic process is not strictly regulated. Therefore, serum 25(OH)D concentration is used to reflect the vitamin D nutritional status in humans (7). Although the Endocrine Society’s 2024 guidelines proposed using different 25(OH)D cutoff values to define vitamin D sufficiency, insufficiency, and deficiency, there is currently no universally accepted reference standard due to a lack of evidence from clinical trials (7). Consequently, the guidelines do not provide explicit alternative thresholds for clinical practice. Against this backdrop, the clinically recognized standard remains derived from the Endocrine Society’s 2011 guidelines, which, based on bone health-related studies, defined serum 25-hydroxyvitamin D levels ≥ 30 ng/mL as sufficient and <20 ng/mL as insufficient (8). Subsequently, multiple authoritative academic organizations and regional guidelines have continued to adopt this threshold, such as the Consensus of clinical application of vitamin D and its analogues (2025) issued by the Chinese Society of Osteoporosis and Bone Mineral Research, Chinese Medical Association, and the Polish Guidelines for the Prevention and Guidelines for Preventing and Treating Vitamin D Deficiency: A 2023 Update in Poland (9, 10). Therefore, while taking into account the directional guidance of the latest guidelines, this study selected serum 25-hydroxyvitamin D < 20 ng/mL as the cutoff for vitamin D deficiency to ensure comparability of the results with mainstream clinical practice and previous studies.

Recently, vitamin D deficiency has been recognized as a global public health issue (11). It is estimated that approximately 65% of the elderly population worldwide has a serum 25(OH)D level below 20 ng/ml, and vitamin D insufficiency is also prevalent among young adults. Numerous epidemiological studies have confirmed that the prevalence of vitamin D deficiency in patients with T2DM is significantly higher than that in the general population (12). Furthermore, multiple studies showed that vitamin D levels were significantly negatively correlated with the risk of T2DM onset, were also negatively correlated with glycated hemoglobin (HbA1c) levels in diabetic patients, and were closely associated with the risk of Diabetic Kidney Disease (DKD), microvascular and macrovascular complications (13–16).

However, in clinical practice, limited by medical resource allocation, testing costs, and patient compliance, systematic screening of vitamin D levels cannot be routinely performed in all patients with type 2 diabetes. In addition, since the optimal serum 25(OH)D level required for disease prevention remains unclear and the cost-effectiveness of large-scale testing is controversial, routine measurement of 25(OH)D in the general population is not recommended (17). At present, the identification of individuals at high risk of vitamin D deficiency mainly relies on clinicians’ empirical judgment, and there is a lack of objective, quantifiable, and individualized prediction tools. On the one hand, this situation may lead to missed diagnoses of high-risk patients, resulting in lost opportunities for early monitoring and timely intervention; on the other hand, low-risk patients may undergo unnecessary testing, further straining healthcare resources (7).

Currently, risk prediction models have demonstrated significant value in research and application within the field of chronic disease management. For example, in elderly patients with osteoporosis, researchers have developed and validated a nomogram model for fall risk based on multiple factors including gender, bone mineral density, and history of falls (18). In the management of elderly patients with psychiatric disorders and stroke, predictive models for suicide risk and oral frailty risk have also been developed and applied, incorporating indicators such as medication status, social support, and nutritional risk (19, 20).

This study aimed to systematically collect clinical data from patients with T2DM and use statistical methods to develop and validate a risk prediction model for vitamin D deficiency suitable for the Chinese T2DM population. It was expected to provide an effective tool for the early clinical identification of high-risk individuals and the implementation of personalized interventions, and to promote the development of refined comorbidity management in diabetes.

## 2 Materials and methods

### 2.1 Study design

This study was a retrospective study conducted on patients admitted to the Department of Endocrinology of a tertiary general hospital in China. Only existing medical records or data were used in this study, which did not involve personal identity or privacy information, pose any risk or harm to patients, and had been approved by the hospital’s Ethics Committee. Therefore, this study was conducted anonymously, and it was not necessary for patients to sign an informed consent form.

### 2.2 Study subjects

A convenience sampling method was used to enroll patients with T2DM who were hospitalized in the Department of Endocrinology between January 2024 and December 2024.

Inclusion criteria: (1) Met the diagnostic criteria for T2DM; (2) Aged ≥ 18 years; (3) Underwent serum 25-hydroxyvitamin D testing.

Exclusion criteria: (1) Pregnant or lactating patients; (2) Patients taking medications that affect vitamin D levels, such as calcium supplements, multivitamins containing vitamin D, or vitamin D supplements; (3) Patients with dietary calcium intake exceeding 1,500 mg/day; (4) Patients with chronic diseases including hypothyroidism or hyperthyroidism, sarcoidosis, tuberculosis, incurable diseases, inflammatory bowel disease, and malignant tumors.

### 2.3 Data collection

The general characteristics of the study subjects included: gender, age, body mass index (BMI), smoking history(SH), diabetes duration (DD), history of hypertension (HTN), and whether complicated with complications (Diabetic Kidney Disease (DKD), diabetic retinopathy (DR), diabetic peripheral neuropathy (DPN), and diabetic peripheral vascular disease (DPVD), and whether using calcium channel blockers (CCB), metformin (Met), sodium-glucose cotransporter 2 inhibitors (SGLT2i), insulin, anticoagulants (AC), and antiplatelet drugs (APDs).

Laboratory and metabolic parameters included albumin (ALB), uric acid (UA), fasting plasma glucose (FPG), triglyceride (TG), triglyceride-glucose index (TyG), total cholesterol (TC), low-density lipoprotein cholesterol (LDL-C), high-density lipoprotein cholesterol (HDL-C), glycated hemoglobin (HbA1c), 25-hydroxyvitamin D [25(OH)D)], estimated glomerular filtration rate (eGFR), and urinary albumin-to-creatinine ratio (UACR). In addition, the season of 25(OH)D sample collection was recorded and categorized as spring (March–May), summer (June–August), autumn (September–November), and winter (December–February).

### 2.4 Sample size

A total of 28 independent variables were included in this study. According to relevant studies, the sample size for a risk prediction model should be at least 5–10 times the number of independent variables (21, 22). In a preliminary sample of 50 cases, the prevalence of vitamin D deficiency was 70%. Considering an invalid sample rate of 10%, the required sample size for this study ranged from 193 to 386 cases. A total of 618 samples were collected in this study, which met the required sample size.

### 2.5 Statistical analysis

All statistical analyses were performed using R software (version 4.5.1). Categorical data were presented as frequencies and percentages, and comparisons between groups were conducted using the chi-square test or Fisher’s exact test. Continuous data with non-normal distribution were expressed as median (M) and interquartile range (P25, P75), while comparisons of ordinal data between groups were carried out using the mann-whitney U test. A two-sided *P* < 0.05 was considered statistically significant. Patients were randomly divided into a training cohort and a validation cohort at a ratio of 7:3. To ensure balanced representation of the outcome variable between the two subsets, stratified random sampling was performed according to vitamin D insufficiency status. All randomization procedures were conducted using R software with a fixed random seed [set.seed(123)] to ensure full reproducibility of the data split. To screen for the predictors most closely associated with the risk of vitamin D deficiency, we simultaneously performed least absolute shrinkage and selection operator (LASSO) regression and multivariate logistic regression analyses. Model development was conducted using R software, and the penalty parameter λ was determined by 10-fold cross-validation to minimize the prediction error. The area under the curve (AUC), sensitivity, specificity, and other relevant indicators were derived by plotting the receiver operating characteristic (ROC) curve. The consistency and calibration degree of the model were evaluated using the Hosmer-Lemeshow (H-L) test and calibration curve, while the clinical effectiveness of the model was assessed via decision curve analysis (DCA). Bootstrap resampling with 1,000 repetitions was conducted for internal validation, and the model’s stability was further assessed using an independent validation set.

## 3 Results

### 3.1 Baseline characteristics of the study subjects and occurrence of vitamin D deficiency

A total of 618 patients with T2DM were enrolled in this study, including 395 males (63.8%) and 223 females (36.1%), with a male-to-female ratio of 1.77:1. The age of the enrolled subjects ranged from 20 to 90 years, with a mean age of 61 years. According to the classification of vitamin D nutritional status, 444 cases (71.8%) were diagnosed with vitamin D deficiency, and 174 cases (28.2%) were non-deficient. As shown in Table 1, there were significant differences between the case group and the control group in the following characteristics: Age, FPG, TG, TC, LDL-C, HbA1c, UACR, Gender, TyG, eGFR, AC, APDs, and DD. Detailed results are presented in Table 1.

**TABLE 1:** Univariate analysis of risk factors for vitamin D deficiency in patients with type 2 diabetes mellitus (T2DM).

Risk factor	Number of cases (*n* = 618)	NON-VDD (*n* = 174)	VDD (*n* = 444)	*P*-value
Age (years)				<0.001
<65	379 (61.3)	74 (42.5)	305 (68.7)	–
≥65	239 (38.7)	100 (57.5)	139 (31.3)	–
Gender				<0.001
Male	395 (63.9)	137 (78.7)	258 (58.1)	–
Female	223 (36.1)	37 (21.3)	186 (41.9)	–
BMI (kg/m^2^)	29.26 [26.03, 32.46]	29.05 [25.63, 33.04]	29.48 [26.06, 32.31]	0.629
DD (years)				<0.001
<5	200 (32.4)	38 (21.8)	162 (36.5)	–
5∼10	94 (15.2)	23 (13.2)	71 (16.0)	–
10∼20	205 (33.2)	65 (37.4)	140 (31.5)	–
≥20	119 (19.3)	48 (27.6)	71 (16.0)	–
SH				0.59
No	436 (70.6)	126 (72.4)	310 (69.8)	–
Yes	182 (29.4)	48 (27.6)	134 (30.2)	–
FPG (mmol/L)	7.36 [5.99, 10.09]	6.69 [5.46, 8.74]	7.71 [6.20, 10.67]	0.001
HbA1c (%)	8.40 [7.20, 10.20]	7.80 [6.80, 9.17]	8.80 [7.40, 10.40]	0.005
TG (mmol/L)	1.40 [0.98, 2.02]	1.27 [0.87, 1.69]	1.44 [0.99, 2.16]	0.003
TC (mmol/L)	4.29 [3.49, 5.12]	4.08 [3.22, 4.93]	4.38 [3.64, 5.20]	0.001
LDL-C (mmol/L)	2.57 [1.93,3.23]	2.40 [1.71,3.05]	2.64 [2.10,3.29]	<0.001
HDL-C (mmol/L)	1.15 [0.97, 1.38]	1.19 [0.98, 1.37]	1.15 [0.96, 1.38]	0.447
TyG	9.04 [8.56, 9.65]	8.83 [8.41, 9.33]	9.18 [8.63, 9.74]	<0.001
ALB (g/L)	40.90 [38.52, 43.38]	40.90 [38.90, 43.30]	40.80 [38.48, 43.40]	0.803
UA (μmol/L)	301.90 [261.00, 374.24]	304.59 [261.69, 389.00]	301.00 [257.99, 365.81]	0.342
eGFR (ml/min/1.73m^2^)	95.83 [74.97, 119.00]	100.40 [76.95, 124.70]	94.26 [74.78, 116.89]	<0.001
UACR (mg/g)				0.019
<300	570 (92.2)	168 (96.6)	402 (90.5)	–
≥300	48 (7.8)	6 (3.4)	42 (9.5)	–
DKD				0.554
No	435 (70.4)	126 (72.4)	309 (69.6)	–
Yes	183 (29.6)	48 (27.6)	135 (30.4)	–
DR				0.688
No	518 (83.8)	148 (85.1)	70 (83.3)	–
Yes	100 (16.2)	26 (14.9)	74 (16.7)	–
DPN				0.061
No	185 (29.9)	42 (24.1)	143 (32.2)	–
Yes	433 (70.1)	132 (75.9)	301 (67.8)	–
DPVD				0.149
No	105 (17.0)	23 (23.2)	82 (18.5)	–
Yes	513 (83.0)	151 (86.8)	362 (81.5)	–
HTN				0.202
No	336 (54.4)	87 (50.0)	249 (56.1)	–
Yes	282 (45.6)	87 (50.0)	195 (43.9)	–
CCB				0.867
No	484 (78.3)	135 (77.6)	349 (78.6)	–
Yes	134 (21.7)	39 (22.4)	95 (21.4)	–
Met				0.205
No	203 (32.8)	50 (28.7)	153 (34.5)	–
Yes	415 (67.2)	124 (71.3)	291 (65.5)	–
SGLT2i				0.788
No	454 (73.5)	126 (72.4)	328 (73.9)	–
Yes	164 (26.5)	48 (27.6)	116 (26.1)	–
Insulin				0.839
No	385 (62.3)	110 (63.2)	275 (61.9)	–
Yes	233 (37.7)	64 (36.8)	169 (38.1)	–
AC				0.002
No	498 (80.6)	126 (72.4)	372 (83.8)	–
Yes	120 (19.4)	48 (27.6)	72 (16.2)	–
APDs				<0.001
No	529 (85.6)	133 (76.4)	396 (89.2)	–
Yes	89 (14.4)	41 (23.6)	48 (10.8)	–
Season				0.339
Spring and winter	372 (246)	99 (56.9)	273 (61.5)	–
Summer and autumn	246 (39.8)	75 (43.1)	171 (38.5)	–

NON-VDD: non-vitamin D deficiency; VDD: vitamin D deficiency.

### 3.2 Screening of risk factors for vitamin D deficiency in patients with type 2 diabetes mellitus

**FIGURE 1:**
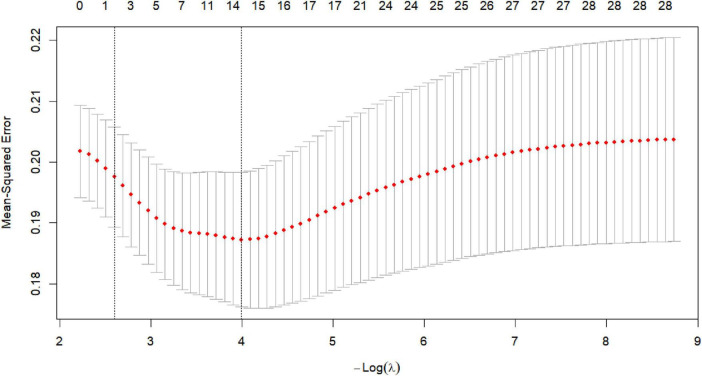
Least absolute shrinkage and selection operator (LASSO) regression analysis: selection of the optimal lambda value.

**FIGURE 2:**
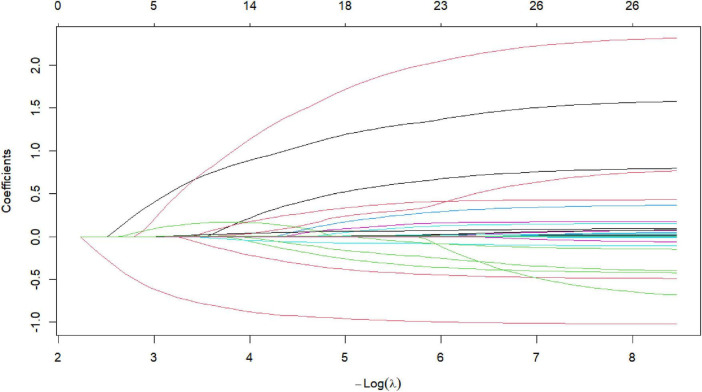
Variable selection by least absolute shrinkage and selection operator (LASSO) regression at the optimal lambda.

Variable selection was performed using Lasso regression combined with 10-fold cross-validation (10-fold CV) in the training set. With λ = 0.0168 (lambda.min, corresponding to the minimum cross-validation error) as the criterion, 14 key predictive variables (with non-zero regression coefficients) were finally retained from the 28 candidate variables. These variables included: Gender (estimate = 0.158), Age (estimate = −0.181), season (estimate = −0.0101), DR (estimate = 0.0385), DPN (estimate = −0.0456), insulin (estimate = 0.0333), AC (estimate = −0.00507), APDs (estimate = −0.00333), UA (estimate = −0.000185), TG (estimate = 0.00350), TyG (estimate = 0.0360), HbA1c (estimate = 0.00777), UACR (estimate = 0.186), and eGFR (estimate = 0.000604). The remaining 14 variables were shrunk to zero and excluded from the final model. Further details are illustrated in Figures 1, 2.

### 3.3 Construction of the nomogram prediction model

**FIGURE 3:**
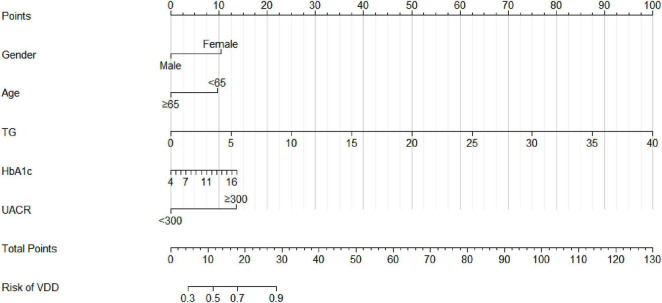
Nomogram for predicting vitamin D deficiency in patients with type 2 diabetes mellitus.

Variables were first screened using Lasso regression, and the selected variables were then included in multivariate logistic regression analysis. The results showed (Table 2) that a total of five variables were identified as independent predictors of vitamin D deficiency. Among them, female was significantly associated with an increased risk of vitamin D deficiency (OR = 3.53, 95%CI: 1.99–6.25, *P* < 0.001). Patients aged > 65 years had a lower risk of vitamin D deficiency (OR = 0.34, 95%CI: 0.19–0.59, *P* < 0.001). In terms of metabolic indicators, TG levels were positively correlated with the risk of vitamin D deficiency (OR = 1.56, 95%CI: 1.15–2.12, *P* = 0.004). The higher the HbA1c level, the higher the risk of vitamin D deficiency (OR = 1.17, 95%CI: 1.03–1.32, *P* = 0.048). In addition, UACR ≥ 300 mg/g significantly increased the risk of vitamin D deficiency (OR = 9.68, 95%CI: 2.58–29.24, *P* < 0.001). Detailed results are presented in Table 2. The nomogram prediction model was constructed by performing a weighted analysis based on the regression coefficients of the independent risk factors, as illustrated in Figure 3.

**TABLE 2:** Logistic regression analysis of risk factors for vitamin D deficiency in patients with type 2 diabetes mellitus.

Risk factor	β	SE	Z	*P*	OR	95% Cl
Gender	1.260	0.292	4.320	<0.001	3.53	1.99–6.25
Age	−1.091	0.289	−3.778	<0.001	0.34	0.19–0.59
TG	0.445	0.156	2.848	0.004	1.56	1.15–2.12
HbA1c	0.119	0.063	1.897	0.048	1.17	1.03–1.32
UACR	2.162	0.619	3.494	<0.001	9.68	2.58–29.24

### 3.4 Validation of the nomogram prediction model

**FIGURE 4:**
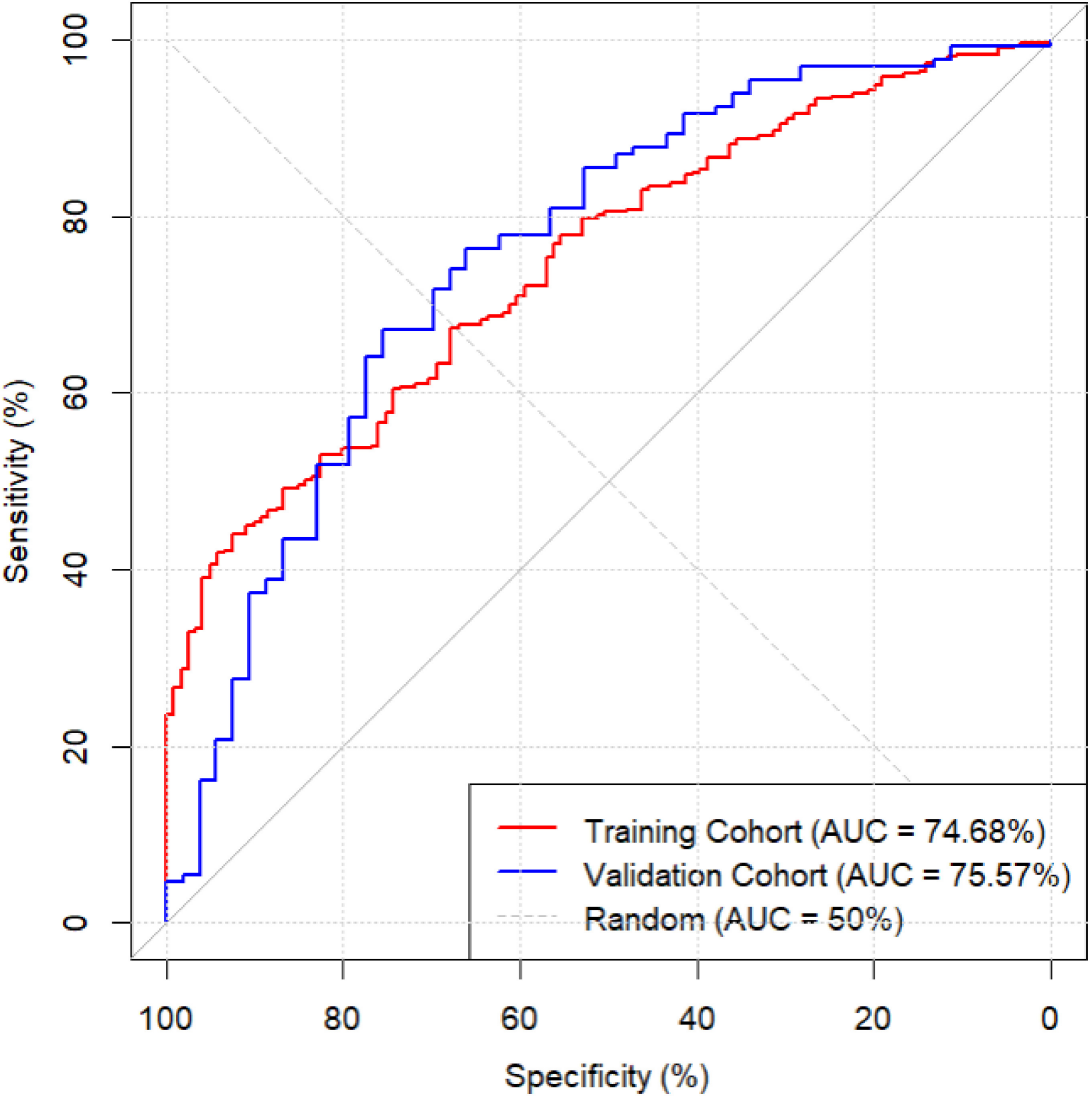
Comparison of receiver operating characteristic (ROC) curves between the training cohort and validation cohort.

The performance of the model was demonstrated by the ROC curve (Figure 4). An AUC value > 0.7 indicated that the model could effectively distinguish patients with high risk of vitamin D deficiency from those with low risk. In the training set, the AUC was 0.7468 (95%CI: 0.6994–0.7942), with a sensitivity of 0.6837, specificity of 0.6364, F1 score of 0.7496, and Youden index of approximately 0.3201. Internal validation was performed via bootstrap resampling with 1,000 replicates. The risk prediction model yielded an AUC of 0.7246 (95% CI: 0.7161–0.7329), sensitivity of 0.6756, specificity of 0.6162, and F1 score of 0.7417, and Youden index of approximately 0.2918. For the validation set, the AUC of the model was 0.7557 (95% CI: 0.6753–0.8362), with an F1 score of 0.7489, sensitivity of 0.6718, specificity of 0.6981, and Youden index of approximately 0.3699. Collectively, these results indicate that the model demonstrated favorable discriminative performance in internal validation. Regarding calibration, the Hosmer-Lemeshow goodness-of-fit test yielded χ^2^ = 9.012 (*P* = 0.436) for the training set and χ^2^ = 6.660 (*P* = 0.672) for the validation set, suggesting acceptable calibration.

**FIGURE 5:**
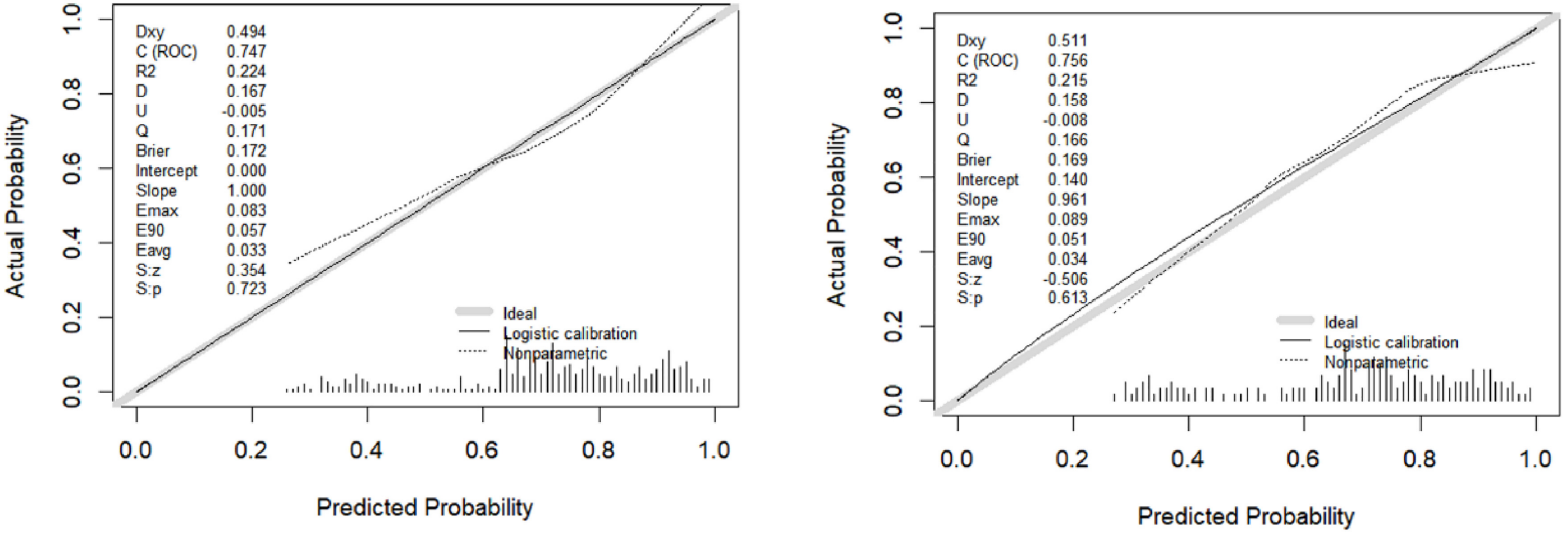
A calibration curves of the nomogram in the training cohort **(A)** and validation cohort **(B)**.

**FIGURE 6:**
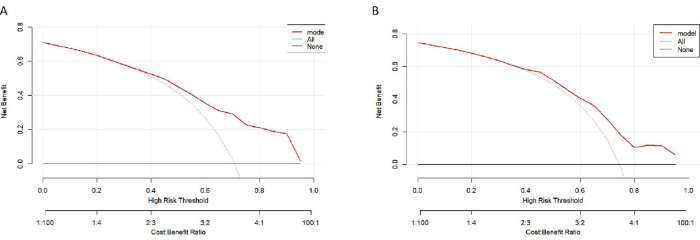
Decision curve analysis (DCA) curves of the nomogram in the training cohort **(A)** and validation cohort **(B)**.

To evaluate the stability of the model, calibration curves of the nomogram were plotted in the training cohort (Figure 5A) and validation cohort (Figure 5B), respectively. The results showed that the slope of the calibration curve was close to 1, indicating good consistency between the predicted values and the actual values. To verify the clinical utility of the prediction model, decision curve analysis (DCA) curves of the nomogram were plotted in the training cohort (Figure 6A) and validation cohort (Figure 6B), respectively. DCA is a commonly used method to evaluate the clinical utility of a model, where the vertical axis represents the net clinical benefit and the horizontal axis represents the threshold probability. The red curve (optimal glm model) represents the net benefit curve derived from the prediction model; the gray curve (full model) represents the net benefit when all patients are assumed to be predicted as high-risk; the black curve (no model) represents the net benefit when all patients are assumed to be predicted as low-risk (no intervention required). If the red curve (optimal glm model) is higher than the gray curve (all patients) and the black curve (no intervention) within most threshold ranges, it indicates that the model has clinical practicality and can generate net benefits under these thresholds. The decision curve analysis showed that the model maintained a positive net benefit even at a threshold probability of 70%, suggesting favorable clinical utility within the current study population.

## 4 Discussion

Patients with T2DM have a high risk of vitamin D deficiency. The results of this study showed that the prevalence of vitamin D deficiency in this population was as high as 71.84%, which was basically consistent with the data (74.14%) reported by Vijay et al. (23) in the Indian T2DM population. Vitamin D deficiency not only increases the risk of osteoporosis in patients with T2DM, but also is closely related to the occurrence of its complications, including an increased risk of cardiovascular events (24), an increased risk of DKD (25), and is also associated with the occurrence of microvascular complications and blood glucose control (26). In this study, a risk prediction model for vitamin D deficiency in T2DM was constructed based on Gender, Age, TG, HbA1c, and UACR.

The results of this study showed that the risk of vitamin D deficiency in females was significantly higher than that in males, which was consistent with the results of previous studies (25, 27). Vitamin D is produced in the skin under the action of sunlight and ultraviolet rays, and only a small part can be obtained from dietary sources. Females often wear clothes that cover their bodies, which affects the synthesis of vitamin D on the skin surface. For example, in Muslim-majority countries, females often wear clothes that cover their entire bodies (28). In China, although females are not required to wear body-covering clothes for religious reasons, the widespread dissemination of theories about sun protection to delay skin aging in recent years has led Chinese females to increase their use of sun-protective clothing and sunscreen, which may be one of the reasons why the vitamin D level of females is lower than that of males.

Notably, we observed a statistical association between age ≥ 65 years and lower risk of vitamin D deficiency, a finding that deviates from conventional expectations. Previous studies generally considered the elderly as a high-risk group for vitamin D deficiency due to declined skin physiological function and reduced outdoor activities. In terms of vitamin D synthesis mechanism, 7-dehydrocholesterol in the skin is converted into previtamin D_3_ under the sun’s ultraviolet (UV-B) light with a wavelength of 290–315 nm. Skin aging markedly impairs this synthetic capacity (29). Skin specimen studies have verified that the concentration of cutaneous 7-dehydrocholesterol drops by more than 50% among individuals aged 20–80 years, and skin vitamin D_3_ production decreases by 13% with each decade of aging (30, 31). Despite age-related synthetic impairment, short-term sunlight exposure can effectively stimulate cutaneous vitamin D synthesis, remaining a vital source of vitamin D for the elderly (32). The positive correlation between age and vitamin D levels observed in this research was consistent with multiple prior findings. A large-sample study involving 14,302 adults across six major Chinese cities conducted by Jiang et al. (27) demonstrated that serum vitamin D levels increased with advancing age. Data from the National Health and Nutrition Examination Survey (NHANES) 2017–2018 cycle indicated that among individuals without vitamin D supplementation, serum 25(OH)D concentrations rose gradually with age, with a more prominent elevation in higher percentiles among older adults (33). It was proposed that some elderly individuals could maintain favorable vitamin D status via natural sun exposure, endogenous cutaneous synthesis and daily dietary intake (33). In this work, the observed age-related trend may be attributed to enrolled population characteristics. Elderly patients with type 2 diabetes presented relatively higher vitamin D levels, closely associated with their health behavioral patterns. Compared with middle-aged and young counterparts, elderly patients generally have longer disease duration, stronger awareness of health management, stricter chronic disease control and more frequent medical follow-ups. They are more inclined to maintain balanced nutritional intake and increase sun exposure voluntarily, thereby elevating vitamin D reserves through both dietary uptake and dermal synthesis. Moreover, potential selection bias may influence the results. The exclusion of participants taking vitamin D supplements in this study serves as a crucial confounding factor influencing the final outcomes. In the study population, participants who actively used vitamin D supplements were mostly individuals with a prior diagnosis of vitamin D deficiency or a high risk of nutritional insufficiency. The exclusion of these subjects screened out a substantial number of elderly samples with low vitamin D levels and reduced the proportion of vitamin D-deficient cases in the elderly group. Such inherent sample selection bias attenuates the well-documented negative association between advanced age and decreased vitamin D levels reported in traditional studies, thereby explaining the positive correlation between advanced age and vitamin D concentrations observed in the present cohort. Restricted by the study design, the regression model failed to fully adjust for variables including dietary fiber intake, duration of sun exposure, cognitive function, and long-term chronic disease burden. Residual unmeasured confounding may distort the genuine association between age and vitamin D nutritional status. In subsequent research, we will establish an independent external validation cohort and repeat relevant analyses to verify the reliability of this age-related correlation.

The increases in TG and HbA1c were significantly associated with an increased risk of vitamin D deficiency, which is consistent with the results of previous studies (34–38). For instance, Vitezova et al. (39) found in a study of 3,240 elderly patients that the risk of hypertriglyceridemia was 31% lower in the vitamin D-sufficient group compared to the deficient group. The potential biological mechanism may be bidirectional. On the one hand, vitamin D deficiency may lead to increased blood glucose and blood lipids by affecting insulin sensitivity, β-cell function, and lipid metabolism; on the other hand, the oxidative stress and inflammatory states associated with hyperglycemia and dyslipidemia may also affect the synthesis and metabolism of vitamin D (40, 41). This suggests that there is a complex interaction between vitamin D levels and glucose and lipid metabolism.

Urinary albumin-to-creatinine ratio ≥ 300 mg/g (i.e., massive albuminuria) is a marker of DKD. This study found that it was closely associated with the risk of vitamin D deficiency (OR = 9.68), which is consistent with the findings of Hong et al. (25), Argano et al. (42). Vitamin D deficiency may accelerate the progression of diabetic kidney disease by activating the renin-angiotensin-aldosterone system (RAAS), promoting inflammation and fibrosis (43). Conversely, impaired renal function can also lead to decreased activity of 1-α hydroxylase in the kidneys, affecting the production of active vitamin D and forming a vicious circle.

Serum 25(OH)D levels are modulated by light-related factors including latitude, climate and season (44). Numerous studies have demonstrated that serum 25(OH)D levels exhibit obvious seasonal variations, with lower levels in winter and spring and peak values in summer and autumn (45–49). Accordingly, the prevalence of vitamin D deficiency is higher during winter and spring (47). A large epidemiological survey involving 14,302 participants from six cities across China reported that the mean serum 25(OH)D level was 22.85 ng/mL in summer and autumn, which was significantly higher than 21.03 ng/mL in spring and winter; the proportion of individuals with vitamin D deficiency was also elevated in spring and winter (27). Consistent findings were obtained from an analysis of 58,834 samples derived from the UDBD-Health database (48). In the present study, serum 25(OH)D levels were slightly higher in summer and autumn (17.25 ng/mL) than those in spring and winter (16.71 ng/mL). The prevalence of vitamin D deficiency was 73.4% (273/372) in spring and winter and 69.5% (171/246) in summer and autumn. However, no statistically significant difference was observed between the two groups. Several factors may account for this discrepancy. First, elderly individuals accounted for a large proportion of the enrolled population, with patients aged ≥ 65 years constituting 61.3% of all subjects, although this study was not restricted to older adults. A previous study of 242 hospitalized patients aged ≥ 60 years found no significant seasonal differences in the prevalence of vitamin D deficiency across four seasons (83.6% in spring, 72.7% in summer, 81.2% in autumn, and 85.0% in winter), which is consistent with our results (50). Second, T2DM directly disrupts vitamin D metabolism. For example, hyperglycemia and aberrantly activated gluconeogenesis inhibit the 25-hydroxylation of vitamin D, thereby reducing circulating 25(OH)D levels (51). Hyperglycemia also upregulates the expression of 7-dehydrocholesterol reductase (DHCR7), a key enzyme for cutaneous vitamin D synthesis, and consequently suppresses vitamin D production (52, 53). Furthermore, patients with type 2 diabetes often suffer from multiple chronic comorbidities. Despite sufficient sunshine duration in summer and autumn, high ambient temperature drives patients to engage in outdoor activities mainly in the early morning or evening. The ultraviolet radiation during these periods is insufficient, which impairs cutaneous vitamin D synthesis (50). These pathological changes attenuate the effects of external environmental factors such as sunlight exposure and season on vitamin D status, leading to non-significant differences in 25(OH)D levels and vitamin D deficiency rates among different seasonal groups in our cohort. Previous studies have indicated that environmental factors such as season and latitude explain less than 20% of the variability in vitamin D levels, suggesting that sunlight exposure is not a decisive determinant of 25(OH)D levels (49). In addition, the relatively small sample size and the failure to collect and adjust for confounding variables including daily sunlight exposure time may also contribute to the lack of significant intergroup differences.

In this study, 25(OH)D was used as the indicator for evaluating vitamin D nutritional status, which is the most widely applied method in clinical practice. For instance, Hu KL et al. (54) adopted 25(OH)D to assess the effect of vitamin D supplementation on live birth rate following in vitro fertilization in women with polycystic ovary syndrome; Ness et al. (55) used 25(OH)D when analyzing the association between low vitamin D levels and proliferative vitreoretinopathy after rhegmatogenous retinal detachment repair; and Assawasaksakul et al. (56) also employed 25(OH)D to evaluate its correlation with mortality and cardiovascular events (including stroke, myocardial infarction, and angina/coronary artery bypass grafting) in patients with systemic lupus erythematosus. However, circulating total 25(OH)D consists of three forms: the free form (approximately 1%), the albumin-bound form (approximately 14%), and the vitamin D-binding protein (VDBP)-bound form (approximately 85%) (57, 58). Notably, VDBP-bound vitamin D is biologically unavailable. Recent studies have indicated substantial interindividual variability in VDBP concentration, and the binding affinity of VDBP to vitamin D is also affected by genetic polymorphisms (59). Therefore, measuring the total 25(OH)D concentration alone to reflect the level of biologically active vitamin D *in vivo* has certain limitations. In recent years, the vitamin D metabolite ratio (VMR), defined as the ratio of 24,25-dihydroxyvitamin D_3_ [24,25(OH)_2_D] to 25(OH)D, has been recognized as a marker that can more dynamically reflect vitamin D metabolism. This ratio represents not only the availability of vitamin D substrate but also its metabolic conversion efficiency, and may provide more physiologically relevant information than measurement of a single metabolite. To date, VMR has been applied to evaluate vitamin D sufficiency and functional vitamin D status in adolescents (59). This study was a retrospective analysis based on routine clinical laboratory data. Limited by the database, we were unable to measure VDBP, free 25(OH)D, 24,25(OH)_2_D, or other related indicators; thus, we could only use total 25(OH)D, the most widely used clinical marker, as the criterion for vitamin D deficiency (60, 61). In future prospective studies, we will establish a more comprehensive evaluation system by incorporating measurements of free 25(OH)D and more precise indicators such as VMR, to further improve the scientific rigor and reliability of the research.

The established prediction model achieved good discriminative performance in both the training and validation sets, with AUC values of 0.7468 (95% CI: 0.6994–0.7942) and 0.7557 (95% CI: 0.6753–0.8362), respectively. The F1 scores were comparable between the two datasets (0.7496 vs. 0.7489), indicating no obvious overfitting. The Hosmer-Lemeshow test showed no statistically significant results for either set (training: χ^2^ = 9.012, *P* = 0.436; validation: χ^2^ = 6.660, *P* = 0.672), which confirmed favorable model calibration and reliable predicted probabilities across different study populations. The model presented moderate sensitivity of 67.18% and specificity of 69.81%, with a Youden index of 0.3699 in the validation set. Nevertheless, all metrics remained stable across the two cohorts, suggesting acceptable generalizability. The slightly higher AUC and Youden index in the validation set further ruled out the possibility of overfitting. Several limitations of this study should be noted. The relatively wide confidence interval of the validation AUC was likely attributed to the limited sample size. In addition, the model had suboptimal sensitivity. Further external validation using large-scale multicenter cohorts and optimization of predictive variables are required to improve its clinical applicability.

## 5 Study limitations

This study has the following limitations: First, this study adopted a single-center retrospective design, which may introduce selection bias and limit its external validity. In addition, all participants were inpatients with severe conditions and numerous comorbidities, and the performance of the developed model among mild outpatient populations remains unclear. Therefore, caution should be taken when generalizing the present findings to outpatient settings, and further external validation studies are warranted. Second, given the retrospective nature of this study, variables including sunlight exposure, nutritional intake, physical activity habits, socioeconomic status and medication profiles were not collected in a standardized manner via the original electronic medical record system, and thus these indicators were excluded from the final prediction model. This limitation can be addressed in subsequent prospective studies through standardized data collection to further improve the predictive performance of the model. Third, due to the study design, it was impossible to determine the causal relationship between each predictor and vitamin D deficiency. Fourth, although internal validation was performed, the model has not been validated in an external multi-center, large-sample population, and its stability and generalization ability need to be further verified.

## 6 Conclusion

In this study, we preliminarily developed and internally validated a nomogram model incorporating Gender, Age, TG, HbA1c, and UACR for predicting the risk of vitamin D deficiency in hospitalized patients with T2DM. Internal validation demonstrated that the model exhibited favorable discriminatory ability, good calibration, and positive clinical net benefit. Moreover, all predictors were routine clinical laboratory parameters that are readily accessible, rendering the model potentially valuable in inpatient settings by providing a quantitative reference for early identification and stratified management of high-risk individuals. Nevertheless, it should be emphasized that the model was constructed based on a single-center inpatient cohort; thus, its direct extrapolation to outpatient or community populations is currently unwarranted. Future multi-center prospective studies and external validations are needed to further evaluate the model’s applicability and stability across diverse healthcare institutions and broader patient populations.

## Data Availability

The original contributions presented in this study are included in this article/supplementary material, further inquiries can be directed to the corresponding author.
